# On the analysis of data augmentation methods for spectral imaged based heart sound classification using convolutional neural networks

**DOI:** 10.1186/s12911-022-01942-2

**Published:** 2022-08-29

**Authors:** George Zhou, Yunchan Chen, Candace Chien

**Affiliations:** Weill Cornell Medicine, New York, NY 10021 USA

**Keywords:** Machine learning, Data augmentation, Cardiac sound analysis, Spectrograms, Convolutional neural network, Cardiology, Healthcare automation

## Abstract

**Background:**

The application of machine learning to cardiac auscultation has the potential to improve the accuracy and efficiency of both routine and point-of-care screenings. The use of convolutional neural networks (CNN) on heart sound spectrograms in particular has defined state-of-the-art performance. However, the relative paucity of patient data remains a significant barrier to creating models that can adapt to a wide range of potential variability. To that end, we examined a CNN model’s performance on automated heart sound classification, before and after various forms of data augmentation, and aimed to identify the most optimal augmentation methods for cardiac spectrogram analysis.

**Results:**

We built a standard CNN model to classify cardiac sound recordings as either normal or abnormal. The baseline control model achieved a PR AUC of 0.763 ± 0.047. Among the single data augmentation techniques explored, horizontal flipping of the spectrogram image improved the model performance the most, with a PR AUC of 0.819 ± 0.044. Principal component analysis color augmentation (PCA) and perturbations of saturation-value (SV) of the hue-saturation-value (HSV) color scale achieved a PR AUC of 0.779 ± 045 and 0.784 ± 0.037, respectively. Time and frequency masking resulted in a PR AUC of 0.772 ± 0.050. Pitch shifting, time stretching and compressing, noise injection, vertical flipping, and applying random color filters negatively impacted model performance. Concatenating the best performing data augmentation technique (horizontal flip) with PCA and SV perturbations improved model performance.

**Conclusion:**

Data augmentation can improve classification accuracy by expanding and diversifying the dataset, which protects against overfitting to random variance. However, data augmentation is necessarily domain specific. For example, methods like noise injection have found success in other areas of automated sound classification, but in the context of cardiac sound analysis, noise injection can mimic the presence of murmurs and worsen model performance. Thus, care should be taken to ensure clinically appropriate forms of data augmentation to avoid negatively impacting model performance.

## Background

Cardiac auscultation has been a core element of the cardiovascular physical exam since the 1800s. Sounds produced by the heart reflect its underlying biology and can cue a trained physician to different heart pathologies such as valvular defects or congenital diseases. However, in recent years, cardiac auscultation has been challenged for its diagnostic utility. The decline in accurate cardiac auscultation is a well-documented phenomenon [1–3]. For example, internal medicine residents in the US made a correct assessment of auscultation findings only 22% of the time [2].

This has spurred an active area of research in developing suitable machine learning models to classify heart sounds based on recorded phonocardiogram (PCG) signals. Many research groups have published a wide variety of machine learning models to this end. Survey of the existing literature reveals that many different feature extraction methods (Mel-frequency cepstral coefficients [4–6], discrete wavelet transform [7–9], tensor decomposition [10], sparse coding [11]) and classification methods (k-nearest neighbors [7], support vector machines [4, 10–12], hidden Markov models [13, 14], recurrent neural networks [15, 16], convolution neural networks [6, 17, 18]), and their different permutations together have been extensively explored.

It is generally accepted that bigger datasets result in better machine learning models [19, 20]. However, real-world clinical applications is limited by the scarcity of labeled clinical data. This scarcity issue can be attributed to several challenges unique to the medical domain, including: the relative paucity of available clinical databases structured for machine learning research, the administrative and logistical hurdles associated with collecting and working with patient data and protected health information due to Health Insurance Portability and Accountability Act (HIPAA) laws and Institutional Review Board (IRB) regulations, and finally the time-consuming and expensive nature of properly annotating health data. The gold standard for validating heart sounds is echocardiogram imaging plus the diagnosis from a cardiologist, both of which are costly to obtain. An additional challenge in creating a machine learning model to classify heart sounds is that heart sounds are not actually recorded and stored anywhere in electronic health records (EHR). Mining EHR databases is not an option, meaning heart sounds must be collected and labeled from scratch, one-by-one. Data acquisition is made even harder in times of public health crises, as we have observed with the COVID-19 pandemic, which resulted in drastic reductions in non-emergency patient volumes in clinics across the world.

Data augmentation is one solution to the legal limitations and constraints around clinical data. Data augmentation is the process of generating *synthetic* data from *real* data, while preserving the class label. In the context of developing machine learning models for heart sound classification, *real* data means heart sounds collected directly from a patient, whereas *synthetic* data means artificial heart sounds generated from *real* heart sounds via various computer-implemented methods.

The major value add of data augmentation for heart sound classification resides in its ability to significantly expand the size of available training data without the onerous task of having to actually obtain and label a large enough volume of heart sounds. An expanded dataset can improve model performance because the new data created from class-preserving transformations can help the model better learn the unique features that constitute the essence of a class, instead of the random variance that is present within each class. Data augmentation combats overfitting and can help the model make better predictions on unseen data.

Data augmentation is necessarily domain specific, as the applied transformations should reflect realistic variations and preserve the underlying features that distinguish different classes from each other. In other words, the data augmentation should ‘make sense’ for the task at hand. Two important constraints unique to heart sound spectrograms must be considered in designing effective data augmentation strategies.

The first constraint, which we will call the “physiological constraint”, is related directly to the phenomenon under study, the heart sound itself. Heart sounds naturally fall within a narrow physiological scope: heart rates are 60–100 beats per minute and the principal frequencies of heart sounds are 20–500 Hz. A healthy heart sound can be deconstructed into four main frequency components: S1 (mitral and tricuspid valve closing), systole (ventricles contracting), S2 (aortic and pulmonic valve closing), and diastole (ventricles relaxing). A pathological heart sound has all the same frequency components. The difference between a healthy heart sound and pathological heart sound is that a pathological heart sound will have additional frequency components such as murmurs from valve stenosis or regurgitation, rubs from pericarditis, S3 gallops(from increased atrial pressure, as seen in congestive heart failure or dilated cardiomyopathy), or S4 gallops(atrium contracting against stiff ventricle caused by hypertension, pulmonary hypertension, ventricular outflow obstruction, or ischemic heart disease). Of note, an additional sound that can be produced by a healthy heart is the physiological splitting of S2 due to delayed pulmonic valve closing. Thus, the “physiologic constraint” is that any data augmentation method must reflect realistic variations of possible heart sounds and also ensure the presence or absence of additional frequency components is preserved for each individual heart sound or else the distinguishing factor between a normal and abnormal heart sound is lost and the class labels lose their meaning.

The second constraint, which we will call the “spectrogram constraint”, is related to the spectrogram image and what it represents. One advantage for using CNN to classify heart sounds is that this converts an audio classification problem into a computer vision problem, which opens the door to the extensive library of data augmentation techniques developed for images. Shorten et al. [21] published a review article surveying the gamut of image data augmentation techniques that have been researched including flipping, cropping, rotation, translations, color space transformations, kernel filters to sharpen or blur images, mixing images, and random erasing. However, not all image data augmentation techniques will translate appropriately. Although spectrograms are images from a data structure point of view, spectrograms and traditional images have a fundamental difference in terms of what information is conveyed along the x- and y- axis. For a traditional image, the axes represent physical distances, while for spectrograms the x-axis represents time and the y-axis represents frequency. Moreover, color also carries a different meaning for traditional images vs spectrogram images. The meaning of color is self-evident for traditional images. For spectrograms, color is an additional dimension that represents decibels, or the loudness and intensity of the heart sound. Thus, the “spectrogram constraint” is that any data augmentation method that operates on the spectrogram as a simple image should correlate with a real-world, physical transformation of the sound.

With these constraints in mind, we evaluate common data augmentation techniques at the audio level, including pitch shifting and time stretching/compressing and noise injection, and at the image level, including horizontal flips, vertical flips, hue/brightness transformations, principal component analysis (PCA) color augmentation, random color filters, and time/frequency masking, for classification of heart sounds based on their spectral image. We include augmentation methods that are consistent with and contradict what would be an effective data augmentation method as predicted by our theoretical considerations discussed above to (1) examine the individual effectiveness of each augmentation technique on heart sound classification and (2) assess the validity of our theoretical framework.

**Fig. 1:**
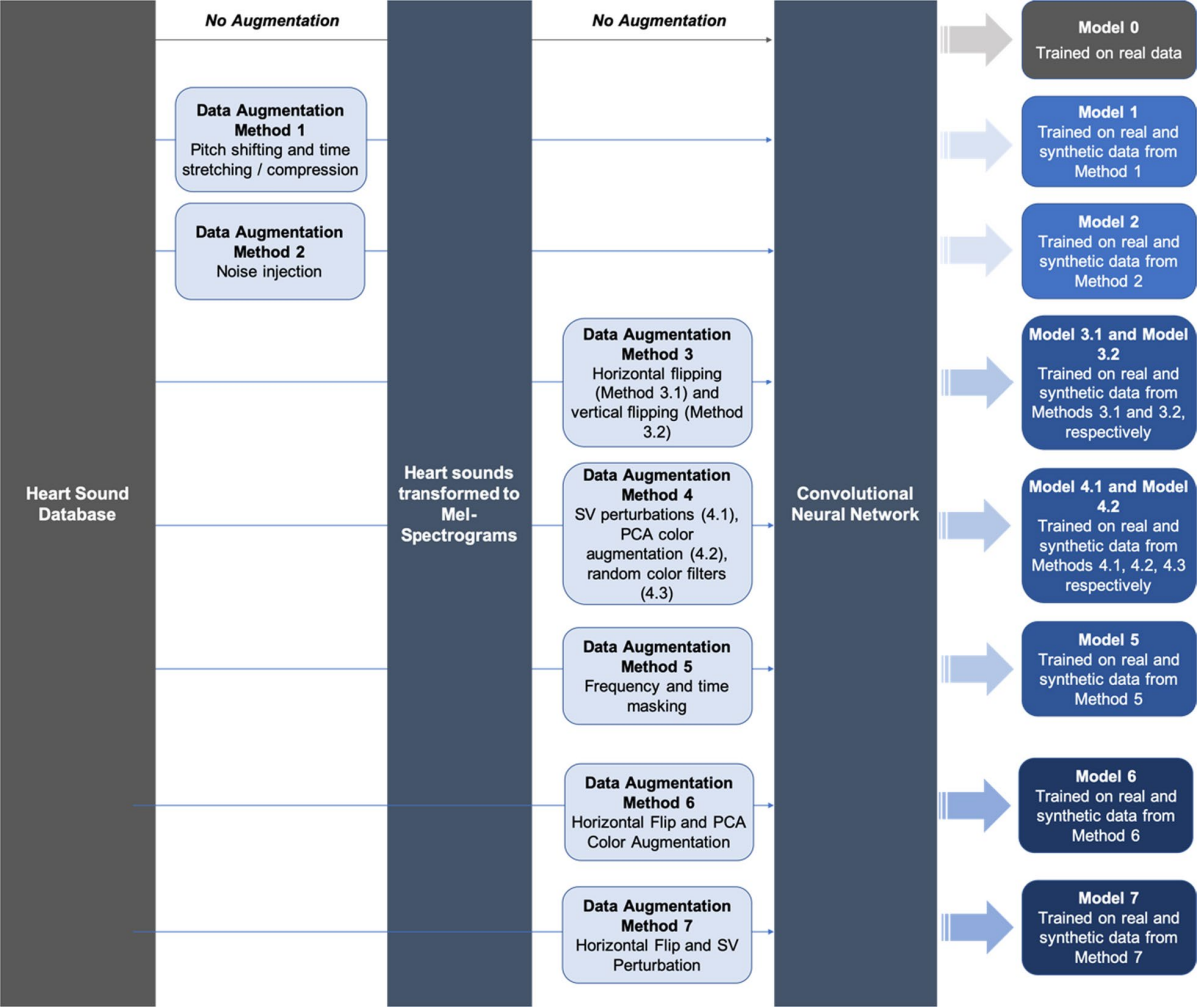
Overview of study design

To study the effects of these data augmentation methods on heart sound classification, we separate our experiments into two phases. The first phase is to establish the baseline performance of our CNN on spectral images of heart sounds. In the second phase, the same CNN is trained on both real and synthetically generated heart sounds. Model performance with and without data augmentation on the same binary classification task is compared. Each individual data augmentation scheme is carried out in a one-to-one correspondence, meaning for every real heart sound, one synthetic heart sound is generated from it. This doubles the size of the dataset available for training, from N to 2N. Figure 1 below shows our study design.

To study the effects of data augmentation on heart sound classification, we established the baseline performance of a machine learning algorithm trained on real heart sound data only (Model 0). We then compared this baseline performance to various models as delineated in the above diagram.

## Methods

### Data

The data in this study was sourced from a publicly available database assembled from the PhysioNet/Computing in Cardiology (CinC) Challenge in 2016 [22, 23]. The directory contains 3,239 recorded heart sounds that range between 5 and 120 s which came from a total of 1,072 subjects. The sounds were compiled by physicians and research teams across seven countries over the course of a decade [22, 23]. Experts in cardiology labelled the heart sounds as either normal or abnormal. Normal sounds are sounds collected from patients with no underlying cardiometabolic conditions. Abnormal sounds are sounds collected from patients with an underlying cardiac pathology, including valvular defects (i.e. mitral prolapse, mitral regurgitation, aortic regurgitation, aortic stenosis and valvular surgery), as well as coronary artery disease [22, 23].

### Pre-processing

In concordance with a previous study on heart murmur identification [24], the raw heart sounds were first processed by a third-order Butterworth filter with a passband of 20–500 Hz, which encapsulates the range of normal heart sound and murmur frequencies [25]. All sounds under 8 s were discarded. Then, the samples were either truncated to 30-s if their length exceeded that limit, or preserved in their entirety if the length less than 30-s. Subsequently, the amplitudes of the signals were normalized according to Eq. 1:

1 $${X}_{norm}= \frac{X}{\mathrm{max}(\left|X\right|)}$$

where *X* refers to the amplitude of the signal to ensure it is standardized across all recordings. Of the remaining heart sounds, 2189 were labeled as normal and the remaining 560 sounds were labeled as abnormal.

### Mel-spectrogram

The samples are windowed using a Hann window of size 512 and hop length of 256. A 512-point Fast Fourier Transform is applied to each window to generate a spectrogram, which depicts frequency over time. The amplitude of each frequency component is encoded in color. The amplitude axis is converted to the dB scale, with the maximum amplitude serving as the reference point and given a value of 0 dB. The frequency axis is transformed onto the Mel scale, which is characterized by Eq. 2,

2 $$Mel=2595*\mathrm{log}(1+\frac{f}{500})$$

where *f* is frequency in Hz.

**Fig. 2:**
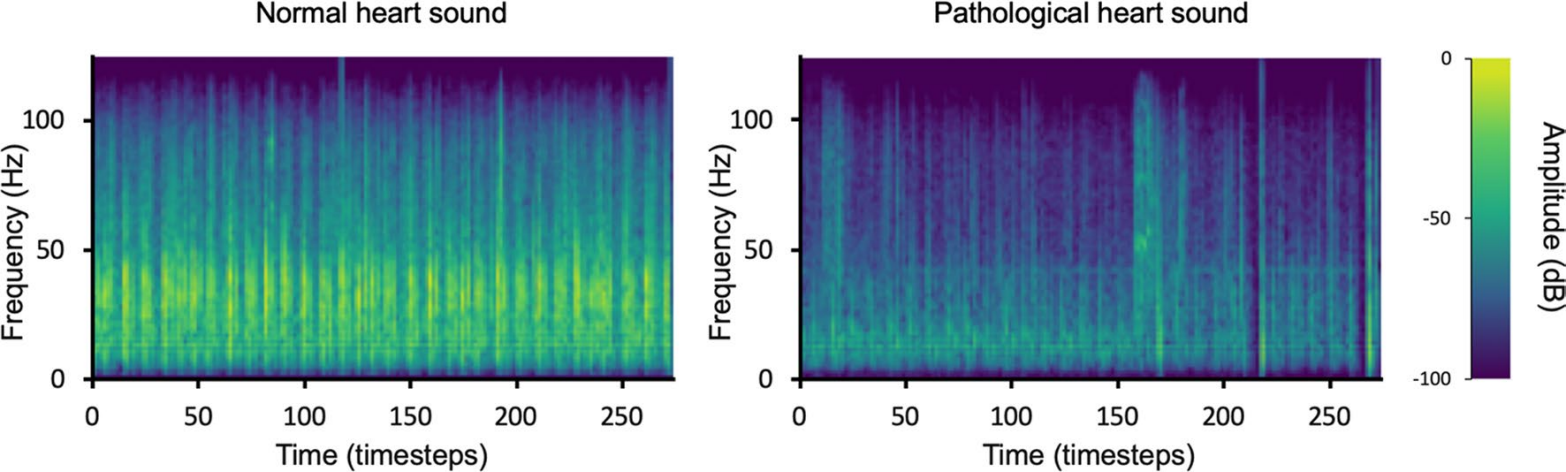
Representative Mel-spectrograms of normal heart sound (left) and pathological heart sound (right)

The resulting Mel-spectrogram images are standardized by rescaling each image to be of size 100 × 180 using bicubic interpolation. Figure 2 shows representative examples of the final Mel-spectrogram images.

### Data augmentation

#### Pitch shifting and time stretching/compression

To create a synthetic heart sound under method 1, each real heart sound is first randomly pitch shifted up or down by *p* semitones, where *p* is a randomly chosen integer between 1 and 10. A semitone is defined as the interval between two adjacent notes in a 12-tone scale. For example, on a musical scale, the interval between *C* and *C#* is one semitone. Then the pitch shifted sound is randomly time stretched/compressed by a factor of *t*, where *t* is randomly chosen from the uniform distribution [0.5, 2.0]. For example, if *t* = 2.0, then a 30 s audio file is stretched to 60 s, or if *t* = 0.5, then a 30 s audio file is compressed to 15 s. The pitched shifted and time stretched/compressed sounds are then converted to Mel-spectrogram images, which are used to supplement the Mel-spectrogram images derived from real heart sounds to train the convolutional neural network.

#### Noise injection

To create a synthetic heart sound under method 2, additive white Gaussian noises (AWGN) are injected element-wise into the original signal. The amplitude of AWGN is modeled as a Gaussian distribution, with $\mu =0$ [26]. The standard deviation of the noise signal is described with the following formula:

$$RMS= \sqrt{\frac{\sum_{i}{x}_{i}^{2}}{n}}$$

Assuming a signal-to-noise ratio (SNR) of 0, the required ${RMS}_{noise}$ can be approximated by ${RMS}_{signal}$. Each element of the noise signal is independently sampled from the distribution $X \sim N\left(\mu ,{\sigma }^{2}\right)$ where $\mu =0, \sigma = {RMS}_{signal}$. The resulting noise signal is summed with the original sample. The synthetic samples are converted to Mel-spectrogram images and combined with the real heart sound Mel-spectrogram database to train the CNN model.

#### Image flip

**Fig. 3:**
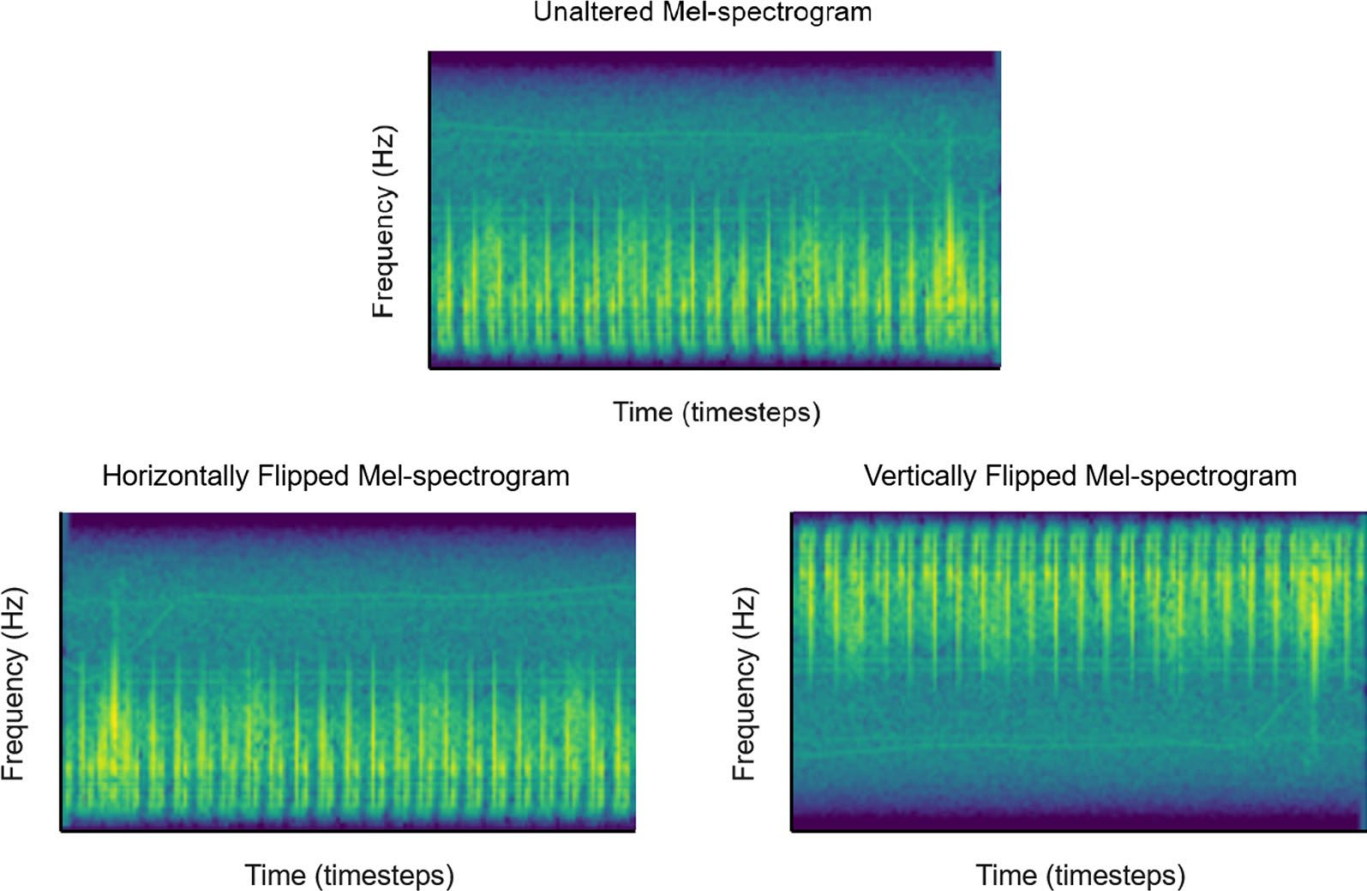
Unaltered Mel-spectrogram (top), horizontally flipped Mel-spectrogram (bottom left), vertically flipped Mel-spectrogram (bottom right)

To create synthetic data under method 3.1, each real heart sound is first converted to a Mel-spectrogram. The images are flipped horizontally, along an imaginary vertical axis that passes through its center, such that a given pixel with coordinate $(x,y)$ will now be situated at $(width-x-1, y)$. Figure 3 displays an example of the transformation. For method 3.2, the images are flipped vertically along a centered horizonal axis, such that a given pixel with coordinates $(x,y)$ will now be situated at $\left(x, height- y-1\right).$ Figure 3 shows illustrative examples of a horizontally and vertically flipped spectrogram image.

#### Color-space transformations

To create synthetic heart sound spectrograms under Method 4, the real heart sounds are first converted into Mel-spectrograms. Then, each image was transformed into their RGB representation, allowing for the extrapolation of other color-space values using pre-established conversion factors and mathematical operations. For example, in an RBG-to-HSV transformation, the red, green, and blue value which range from ([0,255]) for each pixel, is converted into hue ([0°, 360°]), saturation ([0–100%]), and value/brightness ([0–100%]) using the following formulas [27]:

$${R}^{{\prime}}=\frac{R}{255}$$

$${G}^{{\prime}}=\frac{G}{255}$$

$${B}^{{\prime}}=\frac{B}{255}$$

$${C}_{max}=MAX(R{^{\prime}},G{^{\prime}},B{^{\prime}})$$

$${C}_{min}=MIN(R{^{\prime}},G{^{\prime}},B{^{\prime}})$$

$$\Delta = {C}_{max}- {C}_{min}$$

$$H=\left\{\begin{array}{ll}60^\circ x \left(\frac{{G}^{{\prime}}-{B}^{{\prime}}}{\Delta }\mathrm{ mod }6\right), &\quad{C}_{max }=R{^{\prime}}\\ 60^\circ x \left(\frac{{B}^{{\prime}}-{R}^{{\prime}}}{\Delta }+2\right), &\quad{C}_{max }=G{^{\prime}}\\ 60^\circ x \left(\frac{{R}^{{\prime}}-{G}^{{\prime}}}{\Delta }+4\right), &\quad{C}_{max }=B{^{\prime}}\end{array}\right.$$

$$S=\left\{\begin{array}{ll}0, &\quad {C}_{max}=0 \\ \frac{\Delta }{{C}_{max}},&\quad {C}_{max}\ne 0\end{array}\right.$$

$$V= {C}_{max}$$

Within the scope of color space transformations, we explored three modalities of data augmentation. Method 4.1 created new images from saturation and value perturbations. Method 4.2 created new images from Principal Component Analysis color augmentation, a method first introduced in *Krizhevsky* et al. [28]*.* Method 4.3 created new images from applying random color filters.

##### Method 4.1

In Method 4.1, two numbers, ${\alpha }_{brightness}$ and ${\alpha }_{saturation}$, were randomly drawn from a uniform distribution $X \sim U\left(a,b\right)$. Experimentally, it was determined that the ${\alpha }_{brightness}$ would be bounded by a = 0.5 and b = 2, and ${\alpha }_{saturation}$ by a = 0.1 and b = 2. ${\alpha }_{brightness}$ and ${\alpha }_{saturation}$ control the degree of brightness and saturation perturbations, respectively. The merging operation can be described with the following formula:

$$\mathrm{Blending Image }* (1- \alpha ) +\mathrm{ Original Image }* \alpha$$

**Fig. 4:**
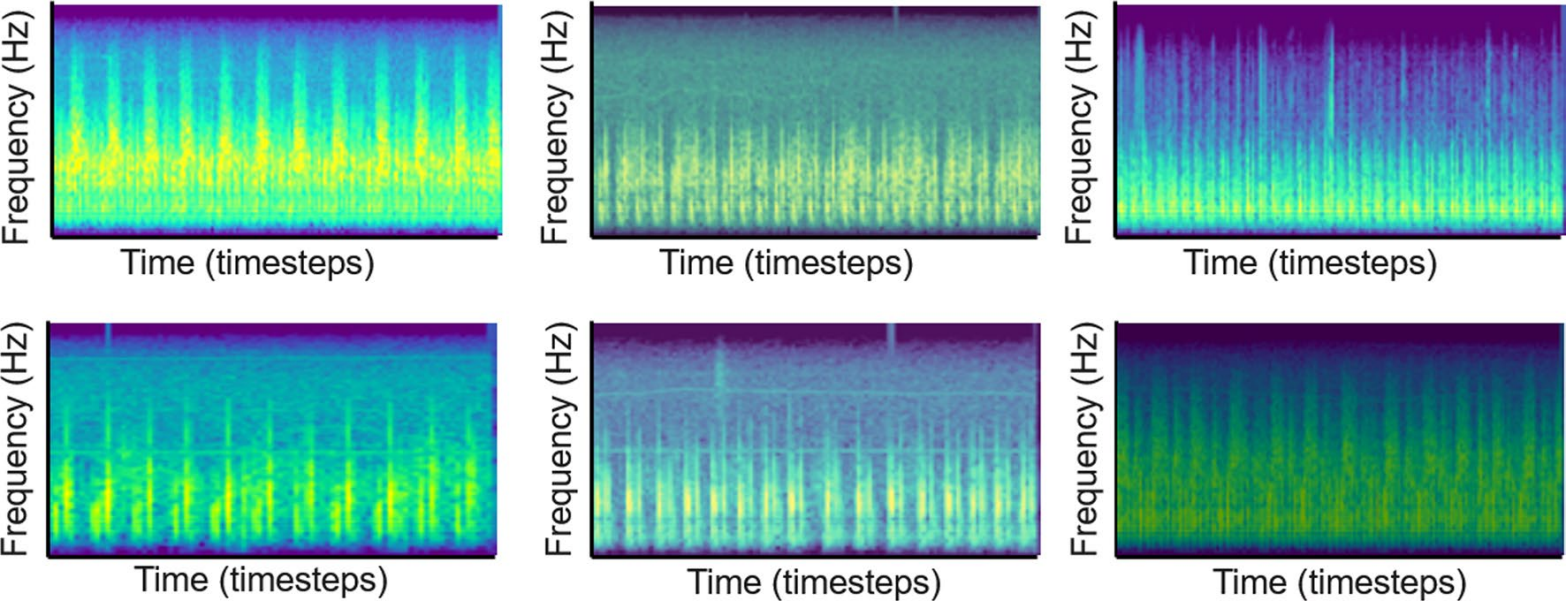
Representative Mel-spectrograms with saturation brightness perturbations

Brightness alterations were achieved by blending the original image with a pure black image of the same dimensions. Saturation alterations were achieved by blending the original image with a grey-scale image of the same dimensions. The two perturbations were applied sequentially to the original image, and the adjustment factors ${\alpha }_{brightness}$ and ${\alpha }_{saturation}$ were redrawn for each input spectrogram. Figure 4 shows spectrograms that have undergone saturation and brightness perturbations.

##### Method 4.2

**Fig. 5:**
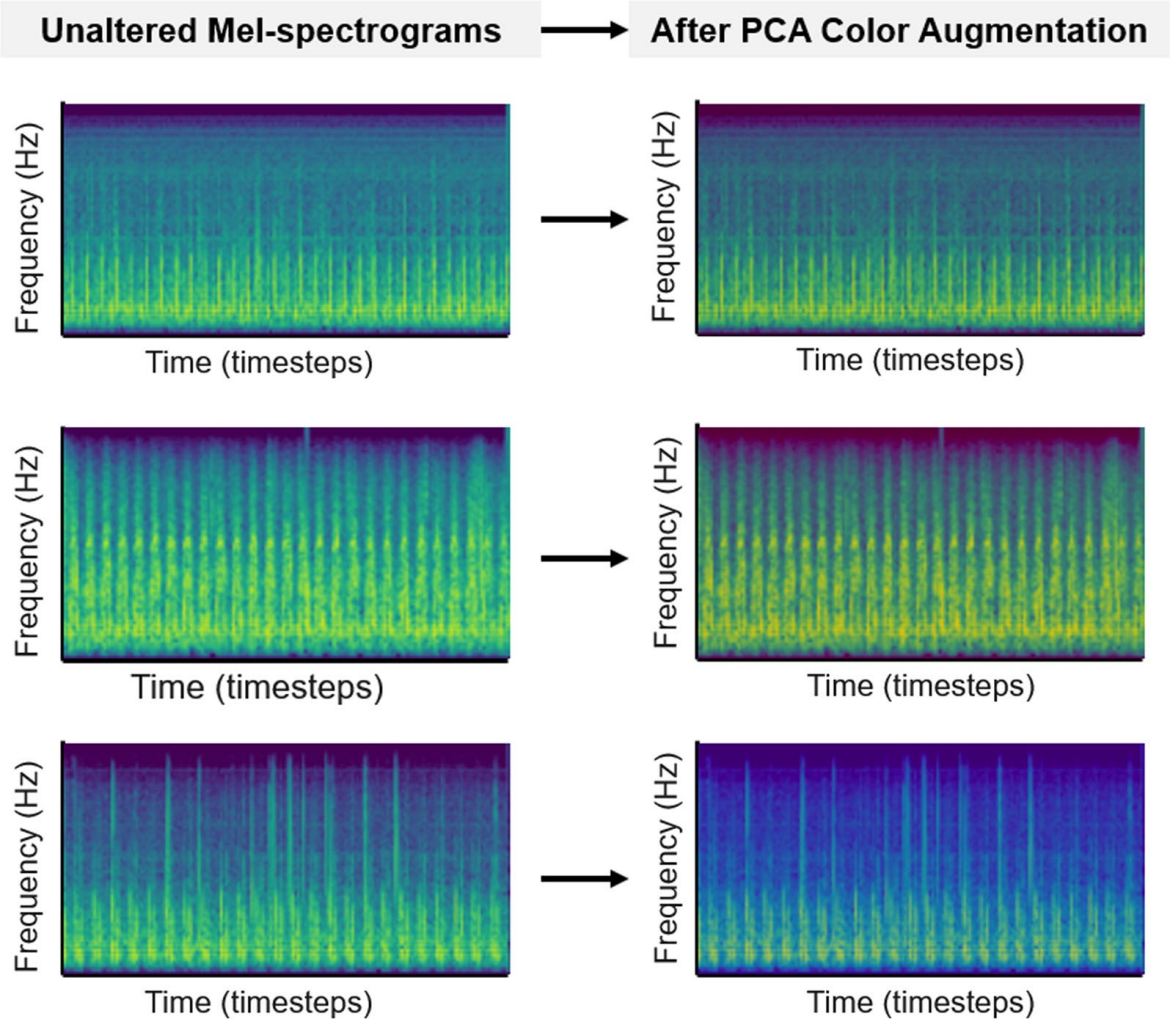
Unaltered Mel-spectrograms (Left), same images after principal component analysis (PCA) color augmentation (Right) (Data Augmentation Method 4.2)

In Method 4.2, as described in Krizhevsky et al. [28], we implemented principal component analysis on the unaltered input images, yielding a sorted set of eigenvectors and eigenvalues that are associated with the 3 × 3 covariance matrix of the RGB color channels. We then drew a random variable $\alpha$ from the normal distribution $X \sim N\left(\mu ,{\sigma }^{2}\right)$, where $\mu =800, \sigma = 10$, and multiplied it to the original eigenvalues. The principal components are scaled by the output from the previous step, and the product is added to the RGB vector of each individual pixel. $\alpha$ is drawn once for each training image. The specific mean and standard deviation values of the perturbation were chosen experimentally, to intentionally produce more pronounced differences in the output images. Figure 5 shows spectrograms that have undergone PCA color augmentation.

##### Method 4.3

**Fig. 6:**
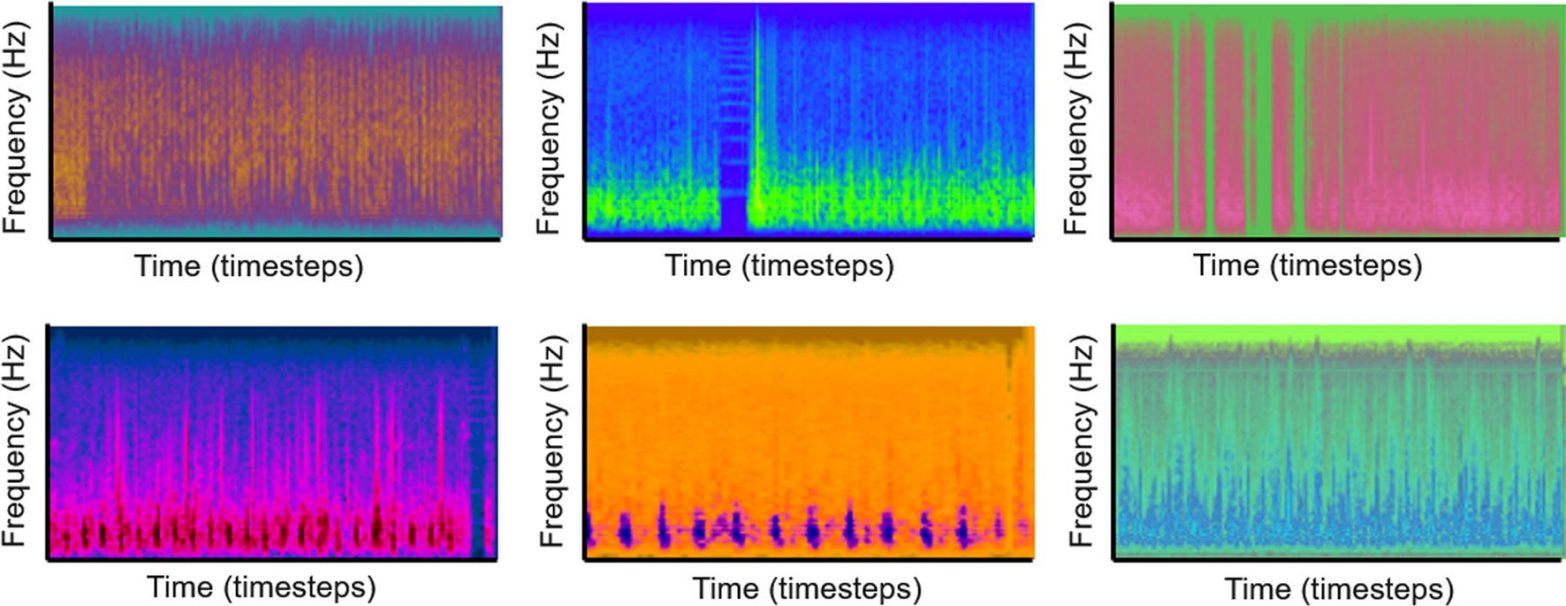
Representative Mel-spectrograms with random color filters

In Method 4.3, we iterated through a library of 150 different color-space conversions using the OpenCV package, effectively generating random color balance perturbations, but preserving the underlying shapes and content of the input images. The transformed Mel-spectrograms are used to supplement the Mel-spectrograms from real heart sounds as additional training data. Figure 6 shows spectrograms with random color filters applied.

##### Time and frequency masks

**Fig. 7:**
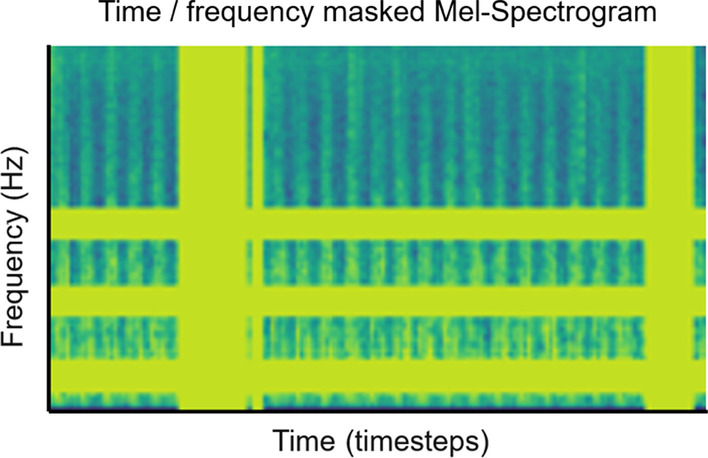
Representative example of time/frequency masked Mel-spectrogram. Three masks, as represented by the yellow bars, are randomly applied in the time domain, and three masks are randomly applied in the frequency domain

To create synthetic heart sound data under Method 5, the real heart sounds are left untouched and converted to Mel-spectrogram images. To the Mel-spectrogram image, three masks are randomly applied in the time domain, and three masks are randomly applied in the frequency domain. In frequency masking, the frequency channels [*f*_0_, *f*_0_ + *f*) are masked, where *f* is randomly chosen from the uniform distribution [0, 20], and *f*_0_ is randomly chosen from (0, *v* − *f*), where *v* is the total number of frequency channels. In time masking, the time steps [*t*_0_, *t*_0_ + *t*) are masked, where *t* is randomly chosen from the uniform distribution [0, 20], and *t*_0_ is randomly chosen from [0, τ − *t*], where τ the total number of time steps. Figure 3 illustrates an example of a transformed Mel-spectrogram. The location of the masks is chosen independently, meaning it is possible for masks to overlap and merge into one larger mask. The transformed Mel-spectrogram images are used to supplement the Mel-spectrogram images derived from real heart sounds to train the convolutional neural network. Figure 7 shows a spectrogram with time and frequency masking applied.

##### Combined horizontal flip and PCA, combined horizontal flip and SV perturbations

In Method 4.6, we augmented the initial images using PCA, and subsequently performed horizontal flip to generate the final transformed spectrograms. In Method 4.7, the initial images were altered using SV perturbation, then flipped horizontally to generate the final transformed spectrograms. The additional spectrograms were used to supplement the Mel-spectrogram images derived from real heart sounds for Model 6 and 7, respectively.

### Convolutional neural network

The resulting Mel-spectrograms are treated as images and used to train a convolutional neural network (CNN) for binary classification. A prior study that explored heart sound classification provided a CNN framework that inspired the basis of the CNN architecture used in this study [29]*.* The convolutional neural network model we built consists of four layers. The first layer is a convolution layer with 32 3 × 3 kernels, each with a stride length of one; the activation function used is a rectified linear (ReLU) activation function.

**Fig. 8:**
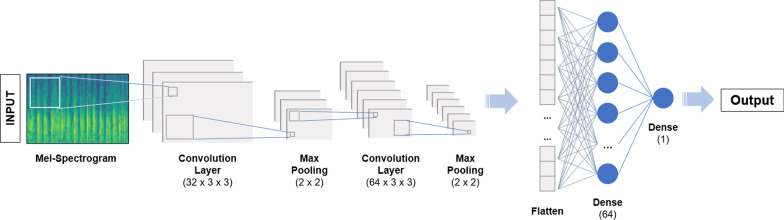
Convolutional neural network structure. Illustration of the CNN architecture employed in our study for heart sound classification

This is followed by a max pooling layer with a filter of size 2 × 2 with a stride length of two. The second layer is a convolutional layer with 64 3 × 3 kernels, each with a stride length of one; the activation function used is a ReLU activation function. Similarly, it is followed by a max pooling layer with a filter of size 2 × 2 with a stride length of two. Padding is not used in any layer. The output from the previous operation is flattened into a one-dimensional feature vector, and then passed to the third layer, a fully connected layer with 64 hidden units. The fourth and final layer is a single neuron with a sigmoid activation function to make the final binary classification. We used the Adaptive Moment Estimation (Adam) optimizer to iteratively improve model performance. Ten epochs are used for training. Figure 8 shows the CNN architecture.

## Results

The folds are created in a consistent way across the different models, meaning each fold for models 0, 1, 2, etc. contains the same set of training/testing data. This serves to limit any potential variability in model performance that would be due to the differences in the training/testing data supplied.

**Fig. 9:**
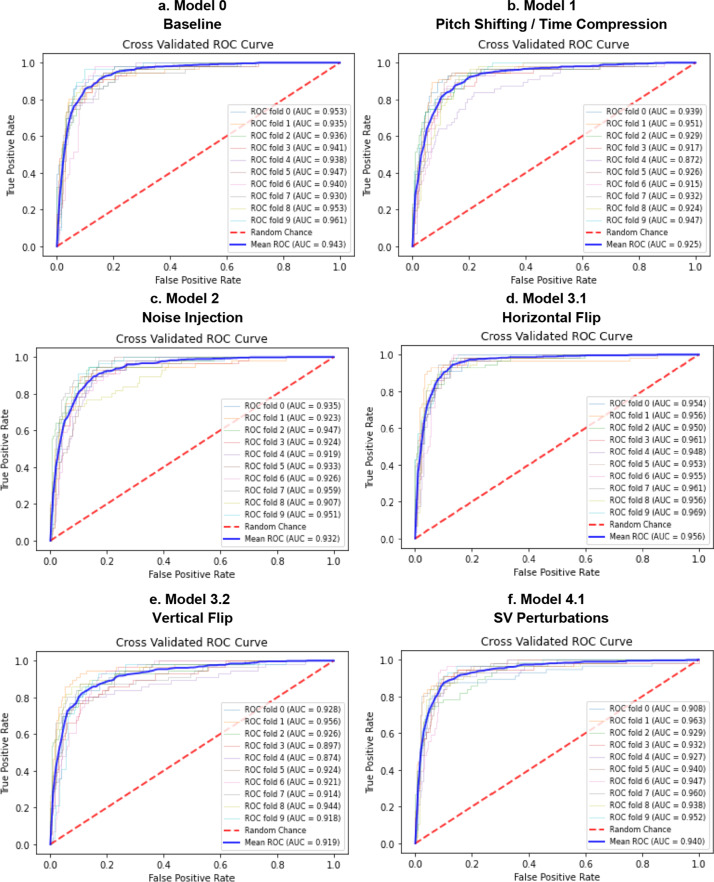

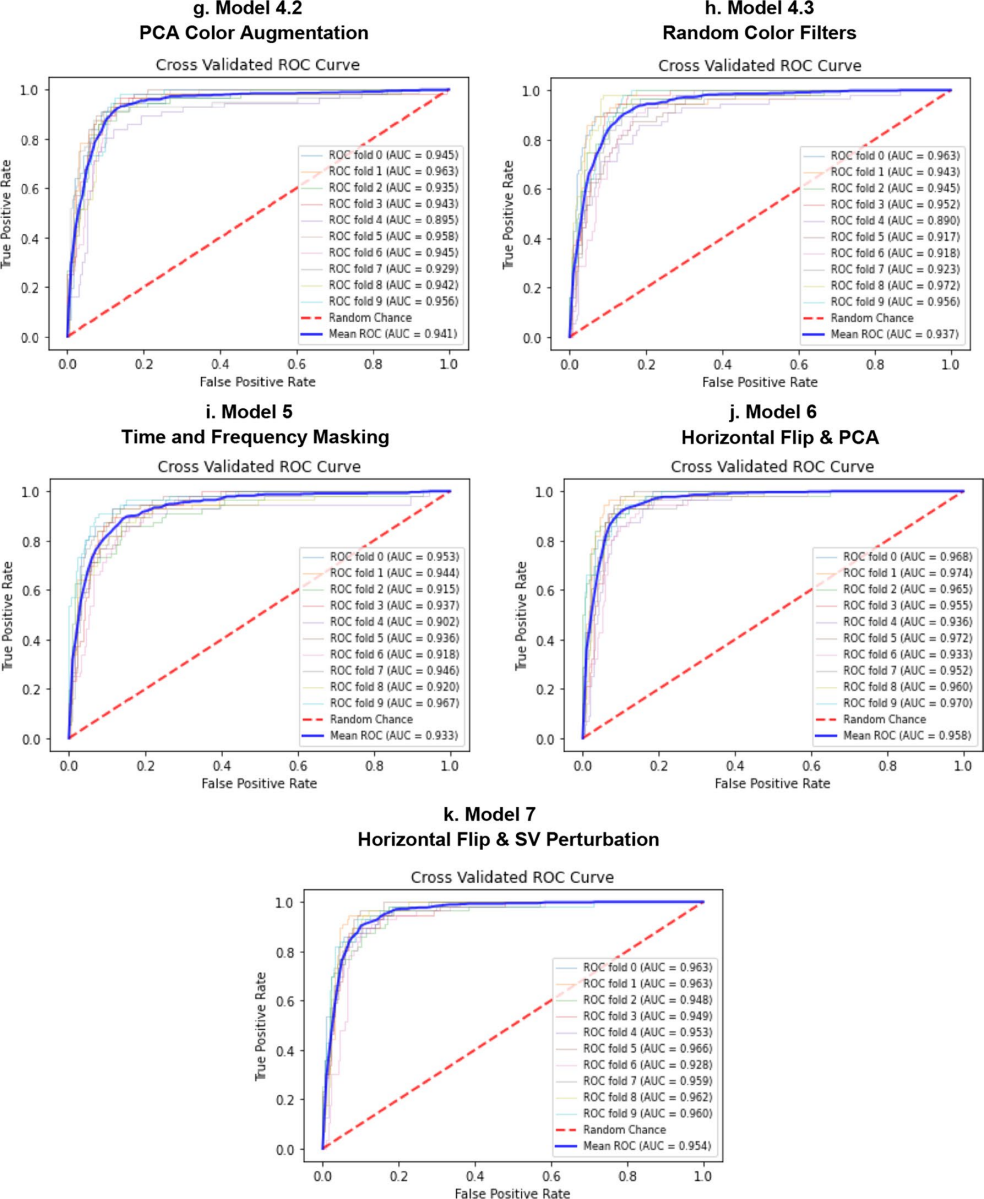
ROC curves for Model 0 (**a**), Model 1 (**b**), Model 2 (**c**), Model 3 (**d**, **e**), Model 4 (**f**–**h**), Model 5 (**i**), Model 6 (**j**), Model 7 (**k**). Comparison of the ROC curve for Model 0, trained on real data only (**a**); ROC curve for Model 1, trained on Mel-Spectrograms of real plus pitch shifted and time stretched/compressed heart sounds (**b**); ROC curve for Model 2, trained on Mel-Spectrograms of real plus noise injected heart sounds (**c**); ROC curves for Models 3.1 and 3.2, trained on real and horizontally flipped Mel-Spectrograms (**d**), and real and vertically flipped Mel-Spectrograms (**e**); the ROC curves for Model 4.1, 4.2, and 4.3, trained on real and saturation/value transformed images (**f**), real and multi-color transformed Mel-Spectrograms (**g**), real and PCA color augmented Mel-Spectrograms (**h**); the ROC curve for Model 5, trained on real and frequency/time masked Mel-spectrograms (**i**); the ROC curve for Model 6, trained on real and horizontally flipped/PCA augmented Mel-spectrograms (**j**); and the ROC curve for Model 7, trained on real and horizontally flipped/SV perturbed Mel-spectrograms (**k**). The dotted red line represents the no-discrimination line

**Fig. 10:**
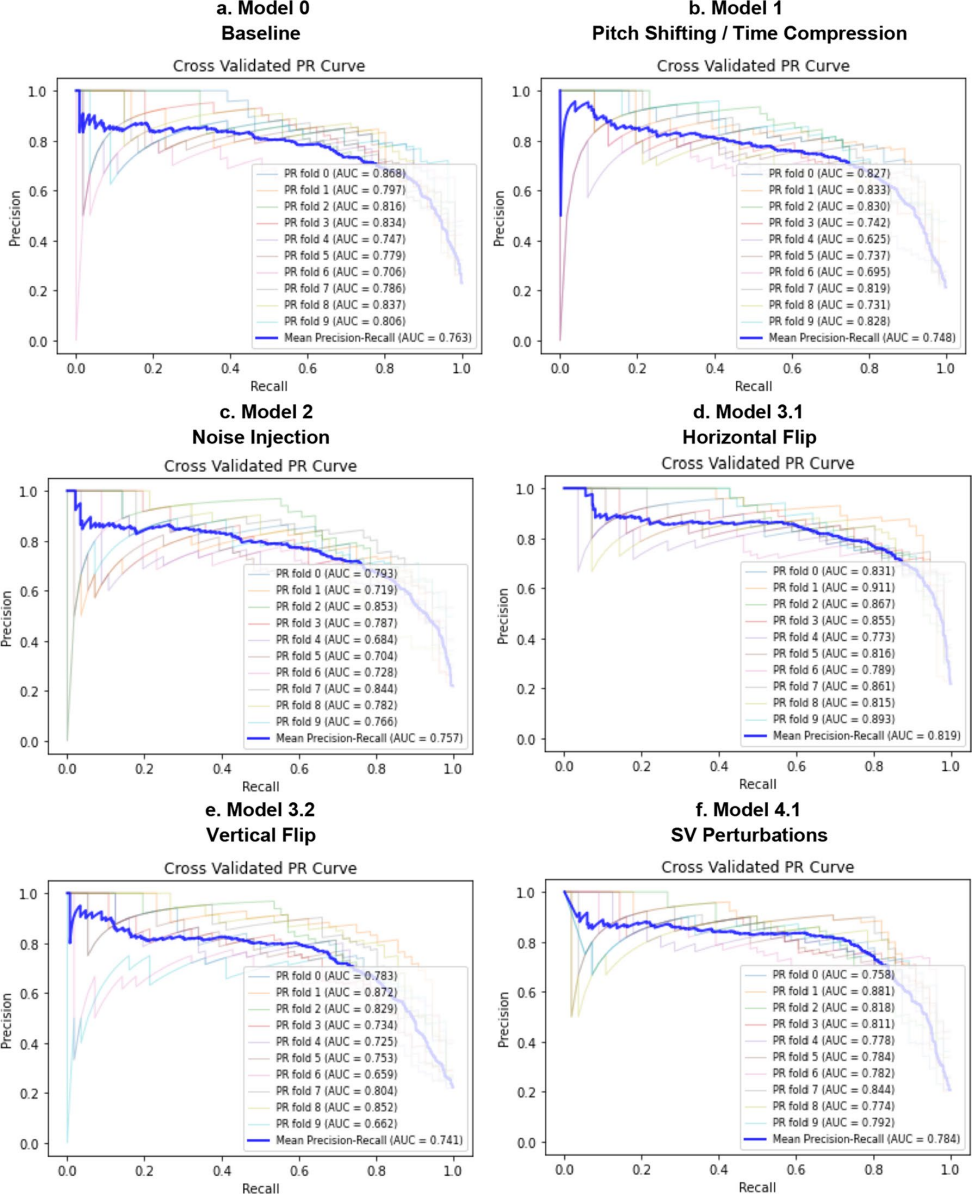

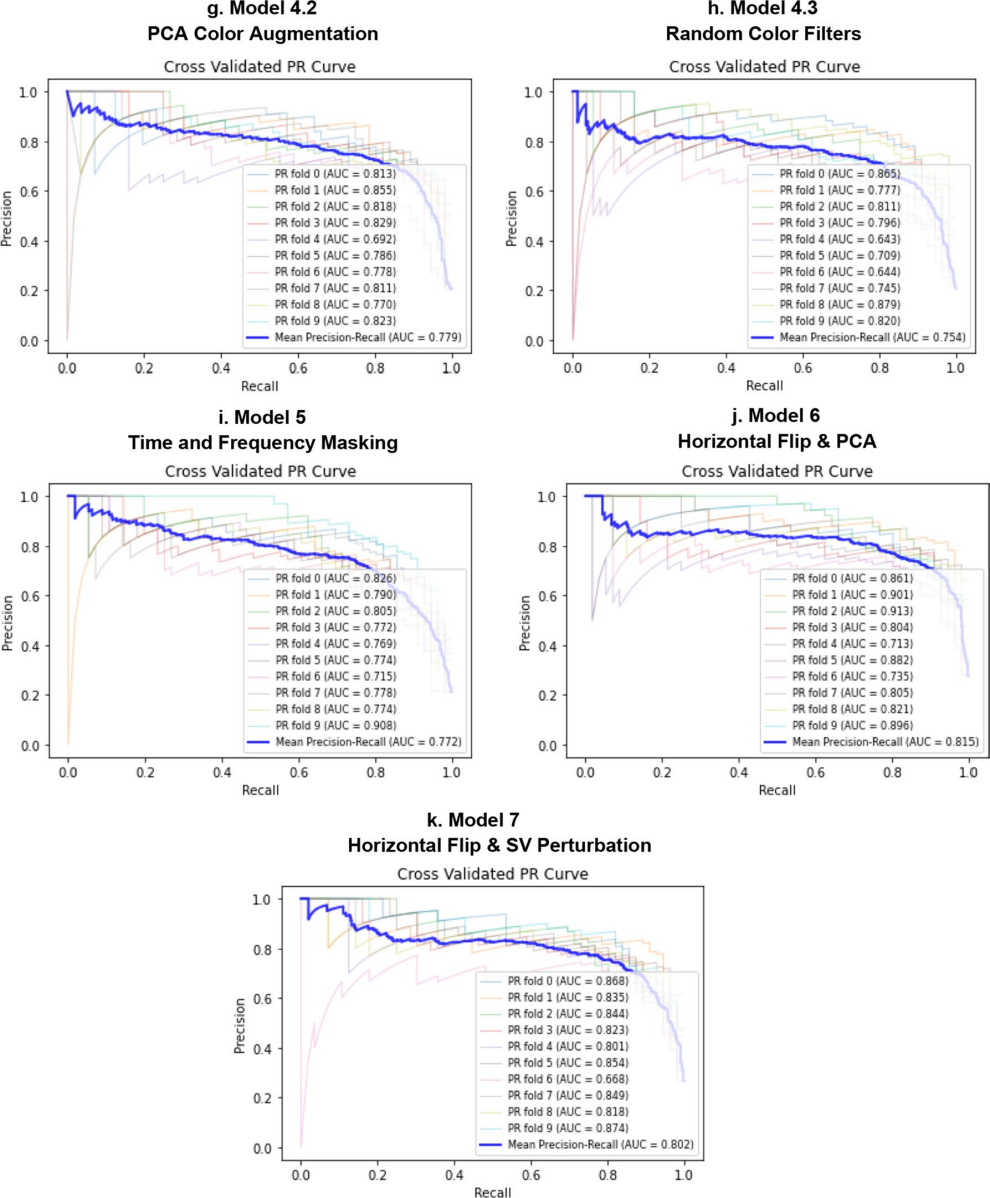
PR curves for Model 0 (**a**), Model 1 (**b**), Model 2 (**c**), Model 3 (**d**, **e**), Model 4 (**f**–**h**), Model 5 (**i**), Model 6 (**j**), Model 7 (**k**). Comparison of the PR curve for Model 0, trained on real data only (**a**); PR curve for Model 1, trained on Mel-Spectrograms of real plus pitch shifted and time stretched/compressed heart sounds (**b**); PR curve for Model 2, trained on Mel-Spectrograms of real plus noise injected heart sounds (**c**); PR curves for Models 3.1 and 3.2, trained on real and horizontally flipped Mel-Spectrograms (**d**), and real and vertically flipped Mel-Spectrograms (**e**); the PR curves for Model 4.1, 4.2, and 4.3, trained on real and saturation/value transformed images (**f**), real and multi-color transformed Mel-Spectrograms (**g**), real and PCA color augmented Mel-Spectrograms (**h**); the PR curve for Model 5, trained on real and frequency/time masked Mel-spectrograms (**i**); the PR curve for Model 6, trained on real and horizontally flipped/PCA augmented Mel-spectrograms (**j**); and the PR curve for Model 7, trained on real and horizontally flipped/SV perturbed Mel-spectrograms (**k**). The dotted red line represents the no-discrimination line

**Fig. 11:**
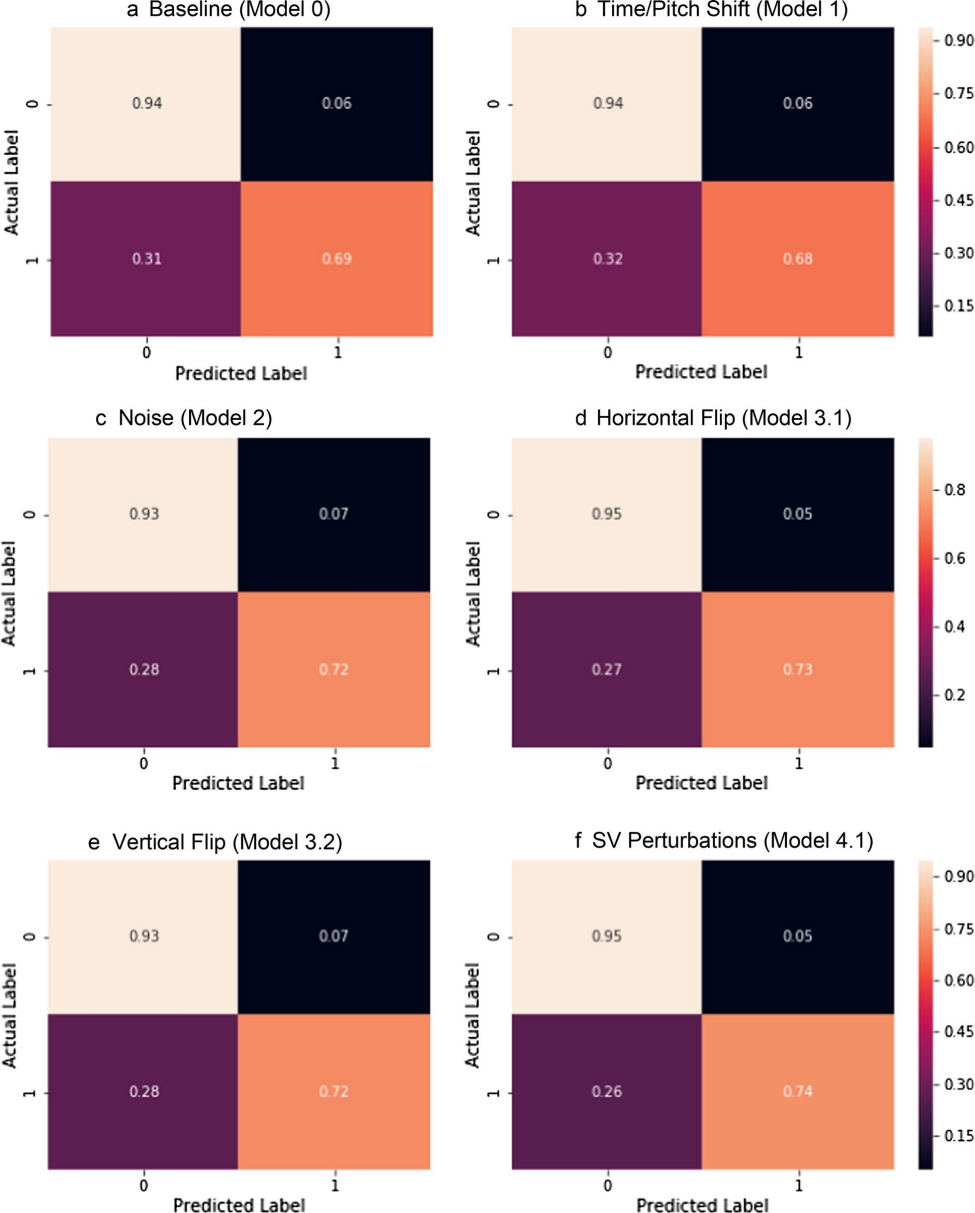

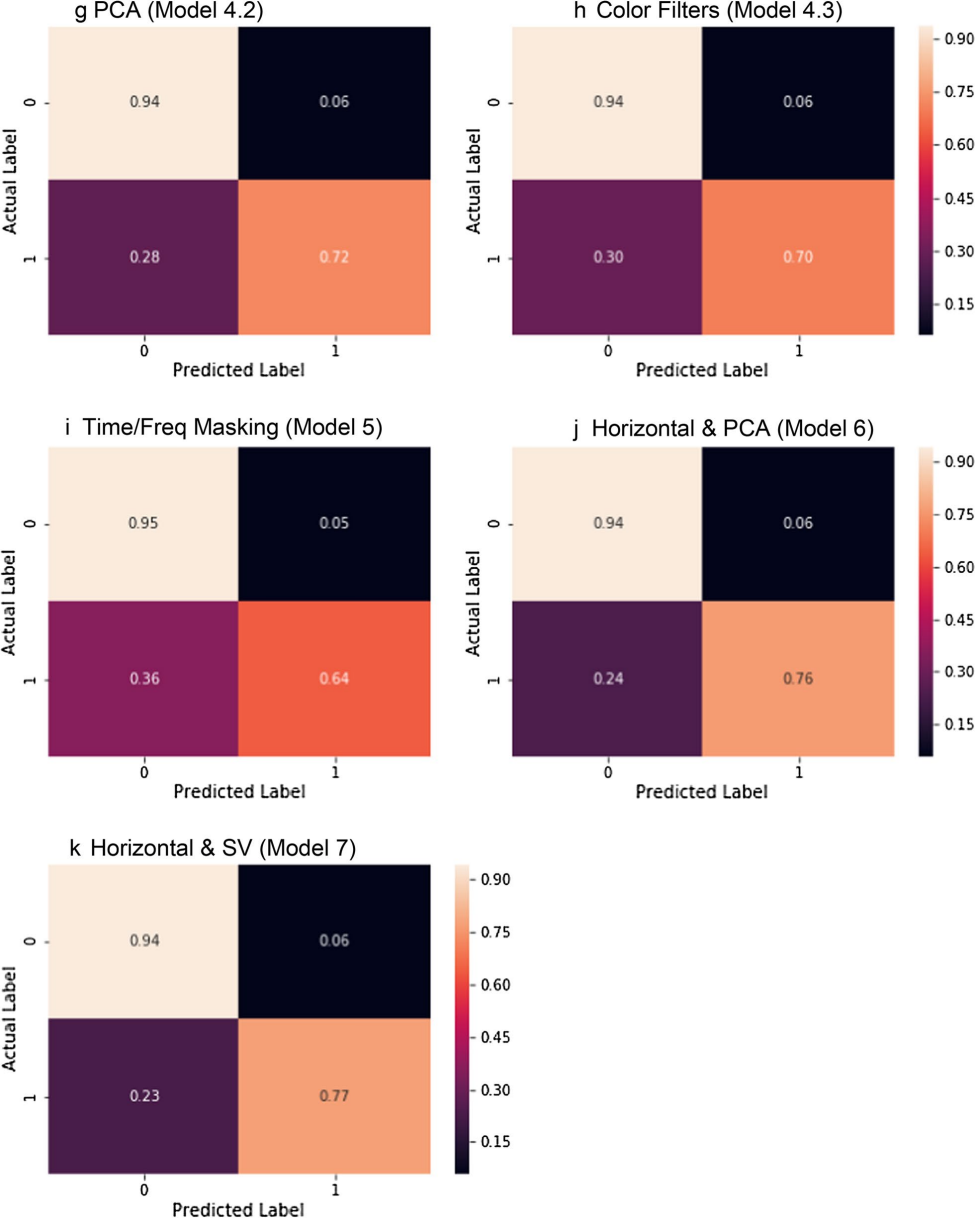
Confusion matrices for Model 0 (**a**), Model 1 (**b**), Model 2 (**c**), Model 3.1 (**d**), Model 3.2 (**e**), Model 4.1 (**f**), Model 4.2 (**g**), Model 4.3 (**h**), Model 5 (**i**), Model 6 (**j**), Model 7 (**k**). 0 represents normal, 1 represented abnormal

Figure 9 shows the cross validated ROC curves for the different models. Figure 10 shows the cross validated PR curves for the different models. Figure 11 shows the confusion matrices for the different models.

Comparison of the confusion matrix for Model 0, trained on real data only; confusion matrix for Model 1, trained on Mel-Spectrograms of real plus pitch shifted and time stretched/compressed heart sounds; confusion matrix for Model 2, trained on Mel-Spectrograms of real plus noise injected heart sounds; confusion matrix for Models 3.1 and 3.2, trained on real and horizontally flipped Mel-Spectrograms, and real and vertically flipped Mel-Spectrograms; the confusion matrix for Model 4.1, 4.2, and 4.3, trained on real and saturation/value transformed images, real and multi-color transformed Mel-Spectrograms, real and PCA color augmented Mel-Spectrograms; the confusion matrix for Model 5, trained on real and frequency/time masked Mel-spectrograms; the confusion matrix for Model 6, trained on real and horizontally flipped/PCA augmented Mel-spectrograms; and the confusion matrix for Model 7, trained on real and horizontally flipped/SV perturbed Mel-spectrograms.

Tables 1 and 2 are numerical summaries of the performance of each model.

**Table 1:** Average performance of each model according to accuracy, specificity at 90% sensitivity, the ROC AUC, and ROC AUC mean difference from baseline model

	Accuracy [95% CI]	Sensitivity [95% CI]	Specificity [95% CI]	ROC AUC [95% CI]	ROC AUC Difference from Baseline [95% CI]
Model 0 *Baseline*	88.7% [87.8, 89.6]	90.1% [89.7, 90.5]	85.1% [82.7, 87.5]	0.943 [0.935, 0.956]	–
Model 1 Pitch/time alterations	88.7% [87.6, 89.8] –	90.2% [89.8, 90.6] ↑	81.3% [75.3, 87.3] ↓	0.925 [0.918, 0.935] ↓	− 0.018 [− 0.031, − 0.004]
Model 2 Noise injection	88.6% [87.7, 89.5] ↓	90.1% [89.9, 90.3] –	82.1% [77.3, 86.9] ↓	0.932 [0.915, 0.943] ↓	− 0.011 [− 0.023, 0.001]
Model 3.1 Horizontal flip	90.7% [89.8, 91.6] ↑	89.5% [89.2, 89.8] ↓	90.4% [88.9, 91.9] ↑	0.956 [0.952, 0.964] ↑	0.013 [0.007, 0.018]
Model 3.2 Vertical flip	88.9% [87.5, 90.3] ↑	90.1% [89.8, 90.4] –	72.8% [64.7, 80.9] ↓	0.920 [0.908, 0.930] ↓	− 0.023 [− 0.037, − 0.008]
Model 4.1 SV perturbations	90.7% [89.6, 91.8] ↑	90.0% [89.6, 90.4] ↓	77.5% [61.3, 93.7] ↓	0.940 [0.933, 0.958] ↑	− 0.004 [− 0.017,0.009]
Model 4.2 PCA color augmentation	89.3% [88.5, 90.1] ↑	90.3% [90.0, 90.6] ↑	87.6% [84.9, 90.3] ↑	0.941 [0.941, 0.958] ↓	− 0.002 [− 0.013,0.008]
Model 4.3 Random color filters	89.1% [87.7, 90.5] ↑	90.0% [89.6, 90.4] ↓	85.1% [80.9, 89.3] –	0.938 [0.912, 0.943] ↓	− 0.006 [− 0.018,0.007]
Model 5 Time/frequency masking	88.7% [87.6, 89.8] –	90.1% [89.7, 90.5] –	83.0% [79.6, 86.4] ↓	0.934 [0.941, 0.956] ↓	− 0.010 [− 0.020,0.002]
Model 6 Horizontal flip and PCA	91.0% [90.0, 92.0] ↑	89.9% [89.7, 90.1] ↓	90.8% [88.8, 92.8] ↑	0.958 [0.949, 0.968] ↑	0.015 [0.006,0.023]
Model 7 Horizontal flip and SV perturbations	90.7% [89.8, 91.6] ↑	90.2% [89.8, 90.6] ↑	91.0% [90.0, 92.0] ↑	0.955 [0.948, 0.962] ↑	0.012 [0.004,0.019]

Specificities were calculated at the threshold value corresponding to about 90% sensitivity for ease of comparison among models

**Table 2:** Average performance of each model according to precision-recall AUC, F1 score, and F1 and PR AUC mean difference from baseline model

	F1 Score [95% CI]	F1 Score Difference from Baseline [95% CI]	PR AUC [95% CI]	PR AUC Difference from Baseline Mean [95% CI]
Model 0 *Baseline*	86.7% [85.7, 87.7]	–	0.763 [0.734, 0.792]	–
Model 1 Pitch/time alterations	84.3% [82.4, 86.2] ↓	− 0.025 [− 0.042, − 0.006]	0.748 [0.703, 0.793] ↓	− 0.031 [− 0.067,0.005]
Model 2 Noise injection	84.6% [83.4, 85.8] ↓	− 0.021 [− 0.035, − 0.006]	0.757 [0.722, 0.792] ↓	− 0.032 [− 0.063, − 0.00002]
Model 3.1 Horizontal flip	87.9% [86.8, 89.0] ↑	0.012 [− 0.00030.024]	0.819 [0.792, 0.846] ↑	0.044 [0.013,0.073]
Model 3.2 Vertical flip	84.5% [83.0, 86.0] ↓	− 0.022 [− 0.038, − 0.006]	0.741 [0.695, 0.787] ↓	− 0.030 [− 0.070,0.0102]
Model 4.1 SV perturbations	87.6% [86.4, 88.8] ↑	0.008 [− 0.006,0.023]	0.784 [0.761, 0.807] ↑	0.005 [− 0.033,0.0425]
Model 4.2 PCA color augmentation	86.4% [85.3, 87.5] ↓	− 0.003 [− 0.014,0.008]	0.779 [0.751, 0.807] ↑	0.000 [− 0.029,0.029]
Model 4.3 Random color filters	85.3% [83.1, 87.5] ↓	− 0.014 [− 0.034,0.005]	0.754 [0.703, 0.805] ↓	− 0.029 [− 0.055, − 0.002]
Model 5 Time/frequency masking	85.1% [83.7, 86.5] ↓	− 0.016 [− 0.031, − 0.001]	0.772 [0.741, 0.803] ↑	− 0.007 [− 0.036,0.023]
Model 6 Horizontal flip and PCA	88.7% [87.5, 89.9] ↑	0.020 [0.004,0.034]	0.815 [0.772, 0.858] ↑	0.036 [− 0.0002,0.0712]
Model 7 Horizontal flip and SV perturbations	88.1% [87.2, 89.0] ↑	0.014 [0.005,0.0213]	0.802 [0.765, 0.839] ↑	0.026 [0.001,0.0507]

## Discussion

In summary, our objective was to identify the optimal forms of data augmentation for the binary classification of PCG signals using their spectral image representation. Our baseline CNN model achieved specificity of 85.1% at 90% sensitivity, a ROC AUC of 0.94, PR AUC of 0.76, and F1 score of 0.87, which makes it comparable to state-of-the-art [30, 31]. As previously discussed, one of the unique challenges of heart sound augmentation is that the generated samples must fulfill certain “physiological constraints” to remain meaningful. More explicitly, the rate, rhythm, and pitch of cardiac sounds are bounded within a narrow range. Values that fall outside of these limits would be unrealistic, and hence detract from the classification. Additionally, the original spectral components of the heart sounds must be maintained to ensure that a normal sound does not become pathological. The presence or absence of frequency components like murmurs, rubs, S3, or S4 gallops should be preserved through these transformations. Secondly, the “spectrogram constraint” stems from the fact that spectrograms and photographs fundamentally convey different information along their respective dimensions. Image data augmentation methods can work for spectral images only if they correlate with realistic physical variations in the sound.

The data augmentation method that satisfied both the “physiological constraint” and the “spectrogram constraint” improved model performance, while all the data augmentation methods that failed to satisfy at least one of the constraints worsened model performance in some respect, experimentally supporting our theoretical framework. We provide a rationale for why each data augmentation method either improved, did not effect, or worsened model performance using our framework below. Our claims of model improvement are based on the 95% confidence intervals of the mean difference between a given model and the baseline model. When the 95% confidence interval of the mean difference includes the value zero, we conclude that there is no statistical difference between the models. When the lower boundary of the 95% confidence interval is greater than zero, we conclude statistically significant improvement. Likewise, when the upper boundary of the 95% confidence interval is less than zero, we conclude statistically significant worsening performance.

Before examining each individual data augmentation technique, the presence of data imbalance in our data set merits a discussion. Our data set contains 2575 normal heart sounds and 664 abnormal heart sounds. Our main strategy for counteracting potential bias introduced by class imbalance was to use a stratified k-fold cross validation, to ensure that the class distribution is maintained in both the training and test sets. We have elected to not utilize any techniques such as under sampling the majority class or over sampling the minority class. Our rationale is two fold. First, we believe these techniques introduces its own set of biases (i.e. if we over sample the minority class we may be overfitting to certain features). Second, we believe a total of 664 abnormal sounds is a sufficiently large enough sample size to build a predictive model and ensure statistical power. Contrast this with a more severely imbalanced data set (i.e. 1000 normal:10 abnormal), where the issue of sample size or statical power is the real issue, not to be conflated with class imbalance. Evidence that our minority class is of sufficient size resides in the fact that our accuracies for all our models range from 88%-91%. If our predictive model simply predicated the majority class every time, the accuracy would be 78%, which means our model is doing more than simply predicating the majority class.

We also note that accuracy, sensitivity, specificity, and ROC AUC, although widely used, may not be the most suitable metric in ascertaining model performance in the context of class imbalance. Our data set has a larger number of negative examples (i.e. normal sounds) and a smaller number of positive samples (i.e. pathological sounds). Given this distribution, a more optimal form of appraisal is precision-recall. Precision is not affected by a large number of negative samples because it measures the number of true positives over the number of samples predicted as positive (true positive + false positives). This makes precision-recall a better metric for evaluating models on an imbalanced dataset compared to sensitivity–specificity because precision-recall measures the ability of a model to correctly identify the positive samples, while sensitivity–specificity measures the ability of a model to distinguish between classes, which is less meaningful when there is a large class imbalance in the dataset. In other words, under the precision-recall paradigm, more weight is given to the accurate detection of positive classes. This rationale makes sense clinically, since it is more costly to miss a murmur than it is to incorrectly classify normal sounds as pathological. This is a general principle in binary classification in medicine, as a false negative is usually worse than a false positive in making a medical diagnosis. Patient safety comes first and foremost to physicians, and there are many supplementary imaging techniques to evaluate suspected murmurs and prevent unnecessary treatment. The burden of dismissing a patient who needs medical attention is much greater than the alternative. We have presented a variety of metrics to capture model performance in our results section, but we will focus on the models PR AUC values in comparing model performance throughout the rest of the discussion section.

The first augmentation method was pitch shifting and time stretching/compressing. Since this augmentation is done at the audio level, the “spectrogram constraint” does not apply. Natural pitch variations reflect different anatomical variations of the heart including differing myocardium wall thickness, body fat/water composition, patient bone/rib structure, and the actual heart size, all of which may lead to variabilities in heart sound attenuation. The data augmentation technique of pitch shifting aims to capture these natural variations. There is also variability in how fast the heart beats. Time stretching and compressing represents heart sounds at different heart rates, such as in tachycardia or bradycardia. Although pitch shifting and time stretching/compressing as data augmentation techniques reflects possible physiological variations, experimentally we see worsening model performance when these data augmentation techniques are applied. At first this seems to contradict our theoretical framework because the “physiological constraint” is supposedly satisfied. However, if we considered that the natural heart sound exists within a very narrow physiological range, it is likely that the upper and lower limits of our pitch shifting, and time stretching/ compressing may have pushed the audio outside the normal physiological range. Thus, the “physiological constraint” was not actually satisfied because our augmentation techniques created sounds that would never exist clinically, which is consistent with the worsening model performance.

The second augmentation method was noise injection. Noise injection has a regularization effect that can improve model performance by reducing overfitting and is a widely used audio data augmentation method for improving model performance. This augmentation is also done at the audio level, so again the “spectrogram constraint” does not apply. Despite the known ability of noise injection for improving model performance, we observe that noise injection actually worsens model performance for heart sound spectral image classification. This can be understood from the fact that the fundamental difference between normal and abnormal heart sounds is that the latter has additional frequency components (murmurs, rubs, S3 gallops, S4 gallops). By definition, noise injection is the act of introducing new frequency components to an audio file. Thus, noise injection is essentially converting normal heart sounds into abnormal heart sounds. Noise injection fails to satisfy the “physiological constraint” because it ruins the distinction that separates normal and abnormal heart sounds.

The third augmentation method is flipping the spectrogram image. Horizontal flipping improved model performance on all three counts, while vertical flipping worsened model performance on all three counts. This is explained by the fact that information conveyed by sound is encoded in the frequency domain, which is represented on the y-axis of spectrogram images. This is an important distinction from traditional images, where the y-axis represents a physical distance. Although vertical flipping has been shown to be an effective augmentation technique for improving model performance on many image datasets such as ImageNet and CIFAR-10 [32] (which consist of images of commonplace objects like dogs, cats, cars, etc.), a vertical flip is not appropriate for a spectrogram image. Transformations of the y-axis of spectrograms would scramble the frequency content of the sound, rendering any meaningful information that was encoded in the sound to be lost. A vertical flip has no physical correlation, and so does not satisfy the “spectrogram constraint.” In fact, the vertical flip worsened model performance the most out of all the data augmentation techniques explored, underscoring the importance of not distorting the y-axis of spectrogram images. Horizontal flipping leaves the frequency axis intact, so it satisfies the “spectrogram constraint”. A horizontal flip alters the temporal relationships of the frequency components, but as discussed above, a normal and pathological heart sound mostly contain the same frequency components (S1, S2, systole, diastole). The major difference is the presence or absence of other frequency components such as murmurs. It is not so much the temporal relationship of these frequency components with each other that help discern a normal heart sound from a pathological one. Thus, horizontal flips satisfy the “physiological constraint” as well, and experimentally we observe that horizontal flips improve model performance the most out of all data augmentation methods explored. Horizontal flipping as a data augmentation technique is most likely unique to heart sound spectral images compared to many other audio classification problems that represent sound as spectral images, owing to the rhythmic nature of heart sounds. In other audio classification tasks such as speech recognition, the temporary relationship of the different frequency components is important, and thus a horizontal flip would most likely hinder model performance.

The next set of data augmentation methods (methods 4.1, 4.2, and 4.3) are various color space transformations. Although these transformations do not distort the frequency axis of the spectrogram, it is important to keep in mind the role of color as an additional dimension in spectrogram images. In a regular photo, color represents the wavelength of light reflecting off an object. In a spectrogram, color represents the loudness/intensity of the signal measured in decibels. Factors that contribute to the natural variation in heart sound amplitudes (i.e. how loud the heart sound is) include the size and position of the heart in the mediastinum, the presence of fluid within or fibrous thickening of the pericardium, and the position and extent of aeration of the lungs. For example, heart sounds are usually loudest at the apex where the heart is in direct contact with the anterior wall of the thorax. Younger patients tend to have louder heart sounds due to elastic and thin chest walls, whereas older patients tend to have quieter heart sounds due to stiffer and thicker chest walls. Heart sounds are louder when the patient is in full expiration, and quieter when the patient is in full inspiration. The data augmentation technique of color space transformations aims to capture these variations. Experimentally, we observe that SV (method 4.1) and PCA (method 4.2) did not statistically improve model performance, while adding random color filters (method 4.3) unequivocally worsened model performance. Neither SV (method 4.1) nor PCA (method 4.2) introduces temporal or spectral distortions to the underlying image, thus satisfying the “spectrogram constraint.” However, specificity at 90% sensitivity post-SV augmentation worsened, likely due to the unconstrained shading changes to the spectrogram, which translates to alterations of loudness/intensity at the audio level. The model is less able to identify “normal” heart sounds due to the unnatural variations in the training set that were labeled as normal. In contrast, incorporation of PCA data in the training set improved specificity at the expense of a minor decrease in ROC AUC. At root, PCA establishes new features, known as “principal components,” from the original dataset. The goal is to compress the initial input dimensionality without compromising the most valuable information that were conveyed. Alterations along these “principal components” accomplish two objectives. First, they enrich the image along the axes of natural variation, which are by definition where the maximum between-sample variabilities exist. Second, since changes are made at the color level, the underlying object invariance is maintained, which preserves the temporal and spectral properties of the original spectrograms. While PCA’s perturbations were derived mathematically, they are still unconstrained by human physiological limits. Therefore, PCA suffers a similar pitfall as SV. Compared to the other augmentation methods aside from horizontal flip, these detrimental effects are arguably much more blunted because the “physiologic constraint” is satisfied to a greater extent. Overall, PCA and SV appear to be the second-best data augmentation methods for cardiac analysis next to horizontal flip.

In contrast to the previous two techniques, random color filters entirely shift the hues outside the scope of our predetermined color-axis (i.e. orange). This may work for images of commonplace objects like cars, which can be observed in a wide variety of colors, but these augmentations are nonsensible for our heart sound spectrograms as they have no associated physical meaning. The spectrogram constraint is severely violated, and experimentally we observe that multicolor filters worsen model performance to the largest degree on all three counts. It is also important to note that in addition to the natural variations in heart sounds amplitudes, changes in amplitude may also reflect clinically relevant information. Pathological conditions such as cardiac tamponade classically lead to diminished heart sounds. Pleural effusions, subcutaneous edema, pneumothorax, and chronic obstructive pulmonary diseases (COPD) such as emphysema would also muffle heart sounds, although in these conditions the heart itself would be considered healthy. Similar to noise injection, alterations in heart sound amplitude could potentially blur the distinction between normal and abnormal heart sounds, which would worsen model performance. Epidemiologically, distant heart sounds from tamponade, pneumothorax, or COPD that is severe enough to muffle heart sounds are much rarer than murmurs. The majority of abnormal heart sounds in our data set are characterized by murmurs rather than distant heart sounds, explaining why amplitude perturbations did not have as much as a deleterious effect compared to noise injections.

The fifth augmentation method is time and frequency masking. Masking induces partial information loss at random points in the time and frequency domain. We surmise that masking has a similar effect to the regularization technique of dropout, where randomly selected neurons are ignored during training. However, in clinical practice, sudden quiescent periods occur in diseases such as AV heart block, cardiac arrest, or sick sinus syndrome. The original labels are preserved, so images that sprung from masking of normal spectrograms are still labeled as normal, despite the introduction of sudden pauses. Hence, masking does not satisfy the “physiologic constraint” and we observe model performance is not improved. Unlike noise injection and similar to amplitude changes, this type of pathological heart sound is relatively rare, thus there is no drastic reduction in performance. This stands in contrast to the state-of-the art results that masking has achieved in automated speech recognition [33], further illustrating the distinction between clinical sound analysis and traditional audio processing.

Compounding data augmentation methods is another way to create additional data diversity. For the sixth and seventh method, horizontal flip was combined with PCA and SV perturbations, respectively. In isolation, the latter two did not consistently improve model performance. In Model 6, cumulative data augmentation achieved higher ROC AUC, sensitivity and F1 score than either horizontal flip or PCA alone. The two methods employed here both provided relatively “physiological” changes that also satisfied the spectrogram constraint. The subsequent outputs fulfilled the previously established framework, and arguably showed the model two types of possible changes through one training set. Model 7 yielded better ROC AUC and F1 score than horizontal flip alone, but slightly worsened specificity. While SV perturbations introduced diversity to help prevent overfitting, it may have pushed some of the horizontally flipped images out of the bounds of normal biology, thus detracting from model learning. The outputs of these models show that concatenating augmentation methods holds promise, but maintaining clinical relevance is still of utmost importance when generating data. For classification problems in medicine, the degree to which synthetic outputs can mimic natural variations in pathology, physiology and clinical features serves as a predictor of their usefulness.

## Conclusions

Our experimental results corroborate our theoretical framework for thinking about heart sound spectrogram classification. Methods that violated the “spectrogram constraint”, such as vertical flipping and applying random color filters, worsened model performance by the greatest extent. Among the methods that did not violate the “spectrogram constraint”, the degree to which the “physiological constraint” was adhered to correlated with how much model performance improved or worsened. Noise injection is not a safe operation because the fundamental distinction between normal and abnormal heart sounds is blurred since the majority of abnormal heart sounds (murmurs, gallops, rubs) are just normal heart sounds with additional frequency components. Amplitude variation (via sensible color space transformations) and masking are also limited by fact that the distinction between normal and abnormal heart sounds are blurred: heart sounds with decreased amplitudes can be found in diseases such as cardiac tamponade, and heart sounds with quiescent periods can be found in disease such as AV block. However, these augmentation methods are less fatal compared to noise injection because epidemiologically these heart sounds are much rarer, explaining why we did not observe a drastic reduction in model performance compared to noise injection. Pitch shifting and time stretching/compressing worsened model performance most likely because the alterations were outside physiological ranges. There is potential for this augmentation method to work but given that heart sounds naturally exist within a narrow physiologic range, future work includes precisely defining these boundaries. Interestingly, horizontal flipping is not actually rooted in any true physiological variation but has proven to be the superior data augmentation method. Horizontal flipping is able to create variation in the data without unnatural variations (such as at the extreme ends of pitch and time alterations) or run the risk of transforming normal sounds into abnormal sounds (such as with amplitude variations or masking). The “physiological constraint” and “spectrogram constraint” can be used as a guide for theory crafting future data augmentation methods for heart sound classification based on their spectral image. Moreover, the ideas behind the “physiological constraint” can be extended to related works seeking to classify heart sounds, while the ideas behind the “spectrogram constraint” can be extended to related work using spectrograms to classify audio.

We recognize several important limitations in our study. While the primary focus of our study was to compare various data augmentation methods for heart sound spectral images, the hyperparameters of the baseline model was selected using a train/test split over the entire dataset. This may introduce potential optimistic bias to the performance metrics, as the model was not optimized using a train/validate/test split. However, the same CNN architecture and hyperparameters were used in every experiment, so any potential bias is maintained throughout, and should not affect our interpretation of the relative differences between data augmentation techniques. In addition, K-fold cross validation does not solve the problem of adapting to a wide range of between-subjective variability. Leave-one-subject-out (LOSO) cross validation would solve this problem but was not used due to the larger computational requirements.

We also note that wavelet transform may achieve a higher baseline classification performance due to better time–frequency localization capacity. Future work includes exploring whether the data augmentation techniques that improved spectral-image based classification will likewise improve performance for wavelet-based classification. Additionally, we hope to explore clinical data classification using other image encoding techniques (i.e. Gramian Angular Field, Markov Transition Field, etc.), and evaluate the effects of data augmentation on their respective model performances.

Despite these limitations, there is value in data augmentation if done correctly, particularly for binary classification of PCG signals, and most likely for other medical classification problems as well. By synthetically generating samples using simple transformations, we can expand on the existing reservoir of patient data, and further enrich the documentation of select pathological conditions, which may be rare in nature and difficult to obtain. Machine learning models are increasingly used to streamline the repetitive processes in healthcare, such as initial screening, preliminary classifications, triage, patient sorting, and specialist recommendations. Data augmentation is a method that has shown utility in improving model performance in cardiac sound analysis and should be further explored in these alternative areas as well. In addition, this study corroborates the idea that models are only as good as the data from which it learns. Disease-appropriate forms of data augmentation are integral to improvements in model performance, and synthetic data is most meaningful when it lies within the scope of human physiology and can accurately mimic clinical findings. Hence, physician input should be considered when creating models, so these tools can be useful and pragmatic both empirically and at the bedside.

## Data Availability

The datasets used and/or analyzed during the current study are available at https://physionet.org/content/challenge-2016/1.0.0/

## Abbreviations

CNN: Convolutional neural networks
AUC: Area under the receiver operating curve
ROC: Receiver operating curve
PCA: Principal component analysis
SV: Saturation/value
PCG: Phonocardiogram
HIPAA: Health Insurance Portability and Accountability Act
IRB: Institutional Review Board
EHR: Electronic health records
COVID-19: Coronavirus disease of 2019
AWGN: Additive white Gaussian noises
SNR: Signal-to-noise ratio
RMS: Root mean squared
RGB: Red/green/blue
HSV: Hue/saturation/value
ReLU: Rectified linear activation function
Adam: Adaptive moment estimation
CIFAR: Canadian Institute For Advanced Research
COPD: Chronic obstructive pulmonary diseases
